# Aerobic exercise during adolescence and anxiety disorders in adulthood: A cohort study using Add Health

**DOI:** 10.1371/journal.pone.0301253

**Published:** 2024-04-11

**Authors:** Whitney S. Córdoba-Grueso, Karla I. Galaviz, Maria A. Parker

**Affiliations:** 1 Department of Epidemiology & Biostatistics, Indiana University School of Public Health, Bloomington, IN, United States of America; 2 Department of Applied Health Sciences, Indiana University School of Public Health, Bloomington, IN, United States of America; SUNY Downstate Health Sciences University, UNITED STATES

## Abstract

**Introduction:**

The prevalence of anxiety disorders, and mental chronic diseases, has increased over the last decade among adolescents. Since aerobic exercise reduces the risk of chronic diseases and stress symptoms, we aimed to examine the association between aerobic exercise in adolescence and anxiety disorders in adulthood.

**Methods:**

Self-reported, publicly available data from 5,114 adolescents who participated in Waves I and IV of the National Longitudinal Study of Adolescent Health (Add Health) was analyzed from 1994–2009. We included US-based individuals aged 16 years on average and observed them for 15 years. Weighted Poisson regression models estimated the association between aerobic exercise in Wave I (1994, baseline) and anxiety disorders in Wave IV (2009, adulthood), adjusting for sociodemographic characteristics and substance use at baseline.

**Results:**

Overall, 639/5,114 (weighted 12.96%) individuals experienced anxiety disorders at baseline. Age and sex differed significantly across all exercise groups (*p*’s<0.001). Aerobic exercise did not significantly protect against anxiety disorders in adulthood: compared to adolescents who did not exercise at all, those who exercised 1–2 times/week had 0.85 times the prevalence of anxiety disorders during adulthood (95% CI = 0.65, 1.12; *p* = 0.25). Those who exercised 3–4 times/week had 0.81 times the prevalence (95% CI = 0.61, 1.08, *p* = 0.15) and those who exercised 5+ times/week had 0.84 times the prevalence (95% CI = 0.63, 1.13, *p* = 0.25) than those who did not exercise at all.

**Conclusion:**

Aerobic Exercise in adolescence did not protect against anxiety disorders in adulthood. More evidence is needed on this association, including using homogeneous measures of exercise and repeated measures methods.

## 1. Introduction

Anxiety disorders are the most prevalent mental health disorders among adolescents in the United States (US). In 2015, 34.1% of adolescents aged 14–18 years old lived with anxiety [1]. A higher prevalence is observed among adolescents aged 12–17 years old, females, and people with lower income or adverse childhood experiences [2–5]. Poor exercise, which is known to increase the risk of physical chronic diseases, might also be associated with chronic mental diseases like anxiety disorders [6]. Understanding the role of exercise on anxiety disorders is important because the trend of daily exercise has decreased over the last 10 years in adolescents, moving from 28.7% in 2011 to 23.2% in 2019 [7, 8], and the prevalence of anxiety disorders has risen from 7.4% in 2009 to 14.66% in 2018 [9].

Regular exercise can modulate anxiety symptoms through several physiological and psychological mechanisms. Psychologically, studies using the Social Cognitive Theory, suggest that regular exercise, particularly moderate intensity and martial arts, increase the self-efficacy of being able to cope with exercise symptoms [10]. Because aerobic exercise causes symptoms common in anxiety-inducing situations (i.e., increased heartbeat and respiratory frequency), regular exercise can also reduce the sensitivity and increase the tolerance for these symptoms in people with a high risk of anxiety disorders [10]. Physiologically, exercise releases endogenous opioids and reduces the reactivity of the sympathetic nervous system and the hypothalamic-pituitary-adrenal axis, which are both activated as a response to stressors in anxiety-inducing situations [10]. Regular physical activity in adolescence is also a significant predictor of physical activity in adulthood [11, 12].

Studies assessing the association between exercise and anxiety among young people report mixed findings. Intervention studies [13] found that exercise, particularly resistance training, reduced anxiety symptoms among youth with baseline depression [14, 15] or baseline CCMD-3 diagnosis of Generalized Anxiety Disorder (GAD) [16]. Exercise interventions were not associated with reductions in anxiety symptoms among young people with eating disorders [17], mild to moderate baseline diagnosis of anxiety/depression [18], or DSM-IV diagnosis of GAD [14]. Observational studies mainly report reductions in anxiety symptoms with exercise. Three studies reported that regular exercise was associated with reduced symptoms of anxiety [19–21], and one more study reported that physical inactivity was associated with experiencing anxiety symptoms [22].

There is more evidence on the association between aerobic exercise and chronic diseases in adults than on the association between aerobic exercise and mental health in adolescents [23–25], and evidence among adults reports that exercise reduces anxiety symptoms [26–28]. Research on the association of exercise and anxiety disorders among adolescents is primarily from randomized controlled trials among people with baseline mental disorders or from cross-sectional studies in global settings. We identified a research gap for US-based studies assessing exercise as a protective factor rather than a treatment, with longitudinal designs and population-level estimates. Building on this prior research, we prospectively examined the association between aerobic exercise and anxiety disorders using a large nationally representative sample. We hypothesized that the risk of an anxiety disorder diagnosis would be higher among adults who did not engage in aerobic exercise throughout their adolescence than those who did.

## 2. Methods

### 2.1 Sample

Data came from the National Longitudinal Study of Adolescent to Adult Health (Add Health), a nationally representative survey of adolescents in grades 7–12 (aged 12–19) in the US. A sample of 20,745 adolescents who completed in-school questionnaires from 80 pre-selected high schools across the nation were recruited and followed for 24 years into adulthood with five waves of in-home interviews from 1994 to 2018, following written parental consent [29, 30]. The average follow-up time for participants was 6 years, with a response rate of 79% at baseline [29]. Add Health used a school-based design [31] with a stratified sampling of non-institutionalized adolescents. Our sample came from the publicly available sample of Add Health, which included 6,504 participants at baseline; 1,390 participants were lost to follow up between 1994 and 2009 [29]. Therefore, 5,114 participants completed both waves 1 (1994–1995) and wave 4 (2008–2009) when our exposure (aerobic exercise) and outcome (anxiety disorders) were assessed, respectively. The Institutional Review Board (IRB) of Indiana University (IRB: #15879) considered this study exempt. The original Add Health study was approved by the IRB of the University of North Carolina.

### 2.2 Measures

The exposure of interest was aerobic exercise which was assessed through self-report from one question on Wave I. The question assessed the frequency of aerobic exercise adolescents performed during the past week as follows: “*During the past week*, *how many times did you exercise*, *such as jogging*, *walking*, *karate*, *jumping rope*, *gymnastics*, *or dancing*?”. Adolescents identified whether they were exercising “not at all”, “1–2 times per week’, “3 or 4 times per week” and “5 or more times per week”.

The outcome of interest was anxiety disorders (Yes/No) which was assessed through a self-reported proxy measure in Wave IV: “*Has a doctor*, *nurse*, *or other health care provider ever told you that you have or had anxiety or panic disorder*?” Anxiety disorders is a term based on the classification of anxiety disorders and panic disorders from the DSM-5 [32].

Covariates of interest were sociodemographic factors such as age in years (range 12–21), sex (Male, Female), race/ethnicity (Non-Hispanic White, Non-Hispanic Black/African American, Hispanic, Asian, American Indian/Alaska Native, and Other), and Household Income (Low <$20K, Middle-Low $20K-49K, Middle-High $50K-74K, and High $75K+) [33]. Past-month tobacco use (Yes/No), past-month binge drinking (Yes/No), as well as past-month cannabis use (Yes/No) were also included as covariates. These variables were defined, respectively, as smoking cigarettes at least once in the past 30 days, drinking 5+ (for males)/4+ (for females) drinks in a row, and having used marijuana at least once in the past 30 days and more than three times in a lifetime [34–37].

### 2.3 Analysis

We used sampling weights to first estimate the prevalence of anxiety disorders and the sociodemographic and substance use characteristics by exercise status. We then conducted a bivariate comparison between aerobic exercise, sociodemographic characteristics and substance use, and anxiety disorder status. We used ANOVA to examine differences in age by exercise status and a chi-square test to examine differences in exercise status by categorical sociodemographic variables (sex, race/ethnicity, income).

Robust Poisson regression models were used to examine the association between aerobic exercise at baseline (Wave I) and prevalent anxiety disorders during adulthood (Wave IV), adjusting for sociodemographic characteristics and substance use at baseline. The selection of the variables to adjust for was based on prior scientific knowledge, considering variables used in previous studies assessing a similar research question [38]. Estimates are presented as unadjusted and adjusted prevalence ratios (PR) with 95% confidence intervals [39, 40]. We used the package “survey” from R Studio (version 4.1.3) for analyses, with Taylor series linearization to account for the complex sampling design. Our missing data assessment revealed only 5 participants missing data on the outcome or exposure and no missing survey weight data [41].

We conducted a sensitivity analysis with different levels of exposure as follows: (Exercising Not at all, 1 or 2 times, 3 or 4 times, and 5 or more times | Exercising Not at all, 1–4 times, and 5 or more times | Exercising Not at all and 1+ times per week). Our findings are robust because the magnitude and direction of the estimates, as well as the significance of the bivariate and univariate analysis, were consistent across different exposure categorizations.

## 3. Results

A total of 5,114 individuals were included in the sample. Individuals were on average 16 years old, half were females (49.5%), 67.3% were Non-Hispanic White, and 37.8% had a Middle-Low Household income (Table 1). Age, sex, and cannabis use differed significantly between exercise groups (Table 1). Tobacco use was present in 27% of our sample, binge drinking in 49% of our sample, and cannabis use in 13%, which was significantly different between exercise groups (Table 1). The prevalence of anxiety disorders was 12.96%. Around 17% of the adolescents did not exercise at all, 33.18% exercised 1–2 times per week, 24.72% exercised 3–4 times per week, and 25.19% exercised 5+ times per week.

**Table 1:** Baseline sociodemographic characteristics of the study population. Data from the National Longitudinal Study of Adolescent to Adult Health (Add Health).

Aerobic Exercise at Baseline (Times per week)						
	Total % (n)	Not at All % (n)	1–2 times % (n)	3–4 times % (n)	5+ times % (n)	*p-value* ^+^
	n = 5110*	n = 823	n = 1638	n = 1283	n = 1366	
**Age, years** ^**a**^	15.87 (0.03)	16.20 (0.07)	15.91 (0.05)	15.69 (0.06)	15.77 (0.05)	**<0.001**
**Sex**						**<0.001**
Female	49.46% (2760)	39.68%	54.11%	51.81%	52.36%	
**Race/Ethnicity**						0.695
Hispanic	4.44% (217)	3.95%	5.05%	3.73%	4.65%	
Non-Hispanic White	67.30% (3037)	69.72%	66.59%	67.12%	66.75%	
Non-Hispanic Black/African American	15.54% (1173)	14.64%	15.58%	15.57%	16.10%	
Asian	2.91% (176)	3.06%	3.00%	3.15%	2.45%	
American Indian/Native American	3.26% (168)	2.79%	0.04%	2.71%	3.43%	
Other	6.53% (316)	5.90%	5.94%	7.71%	6.60%	
**Income, $(USD)**						0.457
Low (<$20K)	18.04% (765)	19.33%	17.64%	18.53%	17.12%	
Middle-Low ($20K-49K)	37.81% (1655)	36.23%	39.47%	39.18%	35.44%	
Middle-High ($50K-74K)	21.39% (925)	21.45%	20.04%	21.77%	22.74%	
High ($75K+)	22.74% (1053)	22.99%	22.83%	20.51%	24.70%	
**COVARIATES**						
Tobacco Use	26.96% (1296)	27.74%	28.09%	25.33%	26.66%	0.503
Binge Drinking	49.16% (2392)	48.81%	48.44%	49.17%	50.31%	0.849
Cannabis Use	12.72% (560)	14.65%	14.05%	11.58%	10.76%	**0.049**
Anxiety Disorder (At wave 4)	12.96% (639)	13.63%	12.83%	12.77%	12.91%	0.958

Percentages and mean estimates are weighted, count estimates are crude.

^a^ Mean (SE)

^+^ANOVA was used to examine differences in age by exercise status. A chi-square test was used to examine differences in exercise status by categorical variables.

* Aerobic exercise counts add up to 5,110 due to missing values in 4 observations.

Table 2 shows the findings of the crude and adjusted Poisson regression models. In the crude model, adolescents who exercised 5+ times/week, 3–4 times/week or 1–2 times/week had respectively 0.95 times (95% CI = 0.74, 1.22, *p* = 0.67), 0.93 times (95% CI = 0.72, 1.21, *p* = 0.62) and 0.94 times (95% CI = 0.74,1.20, *p* = 0.62) the prevalence of anxiety disorders in adulthood compared to those who did not exercise at all. After adjusting for sociodemographic characteristics (age, sex, race/ethnicity, household income) and substance use (tobacco use, binge drinking, cannabis use), adolescents who exercised 5+ times/week, 3–4 times/week or 1–2 times/week were respectively, 0.84 times (95% CI = 0.63, 1.13, *p* = 0.25), 0.81 times (95% CI = 0.61, 1.08, *p* = 0.15), and 0.85 times (95% CI = 0.65, 1.12, *p* = 0.25) the prevalence of anxiety disorders in adulthood compared to those who did not exercise at all. The crude and adjusted models revealed no statistically significant differences in any categories of exercise.

**Table 2:** Poisson regression model evaluating the association between aerobic exercise in adolescence and anxiety disorders in adulthood. Data from the Add Health Study.

Aerobic Exercise at Baseline (Times per week)	Anxiety Disorders (At wave 4)	
	Crude measure of association PR, (95% CI)	Adjusted^a^ measure of association APR, (95% CI)
5+ times (n = 1,366)	0.95, (0.74, 1.22)	0.84, (0.63, 1.13)
3–4 times (n = 1,283)	0.93, (0.72, 1.21)	0.81, (0.61, 1.08)
1–2 times (n = 1,638)	0.94, (0.74, 1.20)	0.85, (0.65, 1.12)
Not at all (n = 823)	Reference	Reference

Note: PR: Prevalence Ratio

^a^ Adjusted for Age, Sex/Gender, Race/Ethnicity, Household Income, Tobacco Use, Binge Drinking, and Cannabis Use.

There were statistically significant associations between anxiety disorders and sex, race/ethnicity, and cannabis use: males were less likely to experience anxiety disorders than females (APR = 0.41; 95% CI = 0.33, 0.51, *p*<0.001), Non-Hispanic Black/African Americans were less likely to have anxiety disorders than Non-Hispanic Whites (APR = 0.50; 95% CI = 0.27, 0.92, *p = 0*.*03*), and those who used cannabis were more likely to have anxiety disorders than those who did not use it (APR = 1.66; 95% CI = 1.27, 2.17 *p*<0.001).

## 4. Discussion

This study examined whether aerobic exercise in adolescence protects against anxiety disorders in adulthood. We found that people who exercised in adolescence were just as likely to experience anxiety disorders in adulthood as those who did not exercise at all. Our findings do not support adopting exercise as a habit in adolescence as an effective strategy to reduce anxiety in adulthood.

In this sample, we found exercise during adolescence did not protect against anxiety during adulthood. Our findings are consistent with those of intervention studies conducted among adolescents with eating disorders or mild to moderate pre-existing anxiety/depression, which reported that exercise did not reduce anxiety symptoms [14, 17, 18]. However, they stand in contrast to many other observational studies that reported reductions in anxiety disorders among adolescents and college-aged adults [20–22] and intervention studies that reported reductions in anxiety symptoms with exercise among adolescents with a baseline diagnosis of depression or anxiety [14–16]. Overall, our findings refuted our hypothesis that over time exercise protects against anxiety disorders.

Women and those who used cannabis had a higher prevalence of anxiety disorders in this study. Previous studies suggest an increased risk of anxiety among people who used cannabis [42] and women experiencing anxiety symptoms due to social norms that condition them to over-worry or to experience thoughts of uncontrollability compared to men, especially in high school settings [43]. Our findings that women had higher odds of experiencing anxiety into adulthood may mean that adolescent women are at higher risk of experiencing this disorder in adulthood. Thus, interventions aimed at preventing anxiety in adulthood should prioritize women and persons with adolescent cannabis use.

Our study had some notable strengths and limitations. A strength of our study is that we used publicly available data that is representative of a large sample of adolescents, collected longitudinally with a high follow-up rate (80.3%) [29]. We used exercise as a multilevel, categorical variable, which allowed us to explore whether exercise exerted a gradient effect on anxiety. Some limitations include the potential for information bias due to our use of self-reported measures of exercise and anxiety disorders and the heterogeneity of the exposure, as exercise measures included different types of activities in a single variable, which limited our ability to explore whether the association differed by exercise type. Additionally, there is potential for misclassification because the questions did not measure other domains of physical activity (i.e.: occupational physical activity and active transport) and they did not measure the intensity nor the duration of physical activity.

Our finding that exercise at baseline was not associated with anxiety in adulthood may be because we did not account for variations in exercise and anxiety disorders throughout time. Therefore, we do not know whether the psychological and physiological effects of exercise occurred frequently enough to exert the reduced anxiety benefits reported in similar studies using the self-efficacy component of the Social Cognitive Theory [10]. We acknowledge some participants might have experienced anxiety at baseline, which could, in turn, have influenced their exercise behaviors. Due to the fact that Add Health did not measure anxiety disorders at wave 1, we could not exclude participants with anxiety at baseline. Confounding could also explain our findings as limited context was provided on other adolescent behaviors that could be associated with exercise or anxiety, such as team sports participation or quality of family and peer relationships [44]. Lastly, the cohort of our study is old enough to be the parents of the current generation of adolescents. Therefore, our estimation of the role of adolescent exercise applies to anxiety disorders among contemporary adults and not current adolescents. The generalizability of our findings is also limited due to the differential attrition by demographics, urbanicity, socioeconomic and immigration status. Add Health has reported higher response rates at wave 4 among US-born individuals, White respondents, females, young individuals, and people with higher socioeconomic status [29].

In conclusion, the findings of our study suggest that exercise in adolescence does not influence anxiety in adulthood. Future studies should use longitudinal designs with repeated measures methods and stratify their findings either by exercise type (if exercise is measured heterogeneously), or by baseline mental health status, as differences in findings from similar studies could be due to differences in the mental health status of their samples. Future studies could also measure exercise with accelerometers instead of self-reported measures and account for other determinants of exercise as covariates, such as participation in team sports with peers or parents, both available in Add Health. Furthermore, future studies could also assess multiple domains of physical activity, such as leisure, occupational, and active transportation. Public Health interventions that aim to prevent or attenuate anxiety disorders in the long term in adults might prioritize female adolescents and combine their strategies with the prevention or cessation of cannabis use.
